# Association between blood eosinophil count and in-hospital mortality among systemic corticosteroids-treated patients with COPD-bronchiectasis overlap: a retrospective cohort study

**DOI:** 10.3389/fphar.2026.1781776

**Published:** 2026-04-29

**Authors:** Wei Liu, Yang Li, Xin Chen, Xuemei Liu, Jianqin Lv, Hongli Jiang

**Affiliations:** 1 Department of Internal Medicine, Division of Pulmonary Medicine, Institute of Integrated Traditional and Western Medicine, West China Hospital, Sichuan University, Chengdu, China; 2 Department of Integrated Traditional Chinese and Western Medicine, Zigong First People’s Hospital, Zigong, China

**Keywords:** bronchiectasis, chronic obstructive pulmonary disease, comorbidity, eosinophil, exacerbation, mortality, systemic corticosteroids

## Abstract

**Background:**

Systemic corticosteroids (SCS) are commonly used for acute exacerbation of COPD (AECOPD), but their effect in patients with COPD-bronchiectasis overlap remains unclear. Whether admission blood eosinophil count (BEC) modifies clinical outcomes in this population is not well-defined.

**Methods:**

This single-center retrospective cohort study included hospitalized AECOPD patients with radiologically confirmed bronchiectasis (2011–2020). Patients were stratified based on admission BEC. The independent associations of BEC with in-hospital mortality and other outcomes in SCS-treated COPD-bronchiectasis overlap patients were rigorously assessed using multivariable logistic regression and propensity score matching analyses.

**Results:**

Among 9,699 AECOPD patients, 1,421 (14.65%) had bronchiectasis. SCS use was more common in patients with greater disease severity and lower baseline BEC. In the overall overlap cohort, SCS was not independently associated with mortality after adjustment. However, BEC significantly modified this relationship. In patients with BEC <300 cells/μL, SCS use was associated with increased in-hospital mortality (adjusted OR 2.05, *P* = 0.038), with the highest risk observed in the very low BEC subgroup (<100 cells/μL) (adjusted OR 8.68, *P* = 0.015), particularly among the elderly (≥70 years) and those with severe radiological bronchiectasis. Conversely, for patients with high BEC level (≥300 cells/μL), SCS use was not linked to increased mortality and was associated with reduced antibiotic treatment.

**Conclusion:**

In this study, BEC appeared to modify the association between SCS and clinical outcomes in hospitalized COPD-bronchiectasis overlap patients. Observationally, SCS was associated with increased mortality in patients with low BEC, especially among the elderly and those with severe bronchiectasis, whereas no such risk was observed in patients with high BEC. These findings are hypothesis-generating and highlight the potential of BEC to inform personalized treatment strategies. However, they require rigorous prospective validation in randomized controlled trials before any clinical implementation can be considered.

## Introduction

Chronic obstructive pulmonary disease (COPD) and bronchiectasis, both chronic airway diseases, frequently coexist and are referred to as the “COPD-bronchiectasis overlap syndrome” (Hurst et al., 2015). The prevalence of radiologically confirmed bronchiectasis among patients with COPD ranges from 4% to 72%, depending on the study population, setting, and diagnostic criteria used (Polverino et al., 2018). Patients with this overlap syndrome typically experience more frequent acute exacerbations, greater lung function impairment, and higher mortality rates compared to those with COPD alone (Martínez-García et al., 2013; Ni et al., 2015; Goeminne et al., 2014). This clinical complexity is driven by recurrent infections stemming from structural airway damage, impaired mucociliary clearance, and chronic bacterial colonization, which collectively intensify inflammatory responses and perpetuate an “infection-inflammation” vicious cycle (Polverino et al., 2018). Despite recognizing these interactions, optimal treatment strategies, particularly concerning corticosteroids use, remain challenging and not yet well-established.

Traditional perspectives caution against the use of inhaled corticosteroids (ICS) in patients with bronchiectasis due to concerns about increased pneumonia risk from localized immune suppression (Polverino et al., 2017). However, emerging evidence suggests that personalizing treatment based on inflammatory phenotypes, rather than relying solely on diagnostic labels, may optimize outcomes. A large cohort study in the UK by Ritchie et al. found that the use of ICS in COPD patients with bronchiectasis did not further increase the risk of pneumonia-related hospitalization (Ritchie et al., 2023). Crucially, blood eosinophil count (BEC) modulated this risk: a BEC >300 cells/μL was associated with a lower risk (OR 0.89), whereas a BEC ≤300 cells/μL increased the risk (OR 1.56) (Ritchie et al., 2023). This suggests that BEC may serve as a biomarker to guide the benefit-risk balance of steroid treatment. Similarly, Chalmers et al. emphasized that the treatment of airway diseases should be based on the type of inflammation, such as Th2-type inflammation, rather than merely the diagnostic label (Chalmers and Shoemark, 2023), with elevated eosinophil levels potentially indicating better responsiveness to steroid treatment and a lower risk of infection.

While the role of ICS in COPD has been extensively studied, the use of systemic corticosteroids (SCS) during hospitalization for acute exacerbations of COPD (AECOPD) in patients with concomitant bronchiectasis remains poorly defined and lacks consensus. SCS are commonly administered for their anti-inflammatory effects during exacerbations, yet they may further compromise immune function and increase infection risks, particularly in individuals with chronic bacterial colonization in bronchiectasis (Everaerts et al., 2018). Although studies have evaluated BEC during COPD exacerbations (Bhatt et al., 2023), whether BEC measured at admission can predict differential responses to SCS treatment in hospitalized COPD-bronchiectasis overlap patients, particularly for critical outcomes such as in-hospital mortality, mechanical ventilation need, and length of stay, remains unexplored.

This study aimed to retrospectively analyze, using clinical data from West China Hospital, Sichuan University, the influence of BEC on the outcomes of SCS therapy in hospitalized AECOPD patients with radiologically confirmed bronchiectasis. We hypothesized that a higher BEC at admission would be associated with better clinical outcomes and lower complication risks following SCS administration, thereby offering evidence to inform personalized treatment strategies for this complex patient population.

## Materials and methods

### Study design and data sources

This hospital-based retrospective cohort study utilized data from the Clinical Big Data Platform for Scientific Research (CBDPSR) at West China Hospital, Sichuan University, a tertiary referral center in Southwest China that integrates de-identified electronic health records from over 1.5 million patients, including demographics, diagnoses, treatments, imaging, and laboratory data (Yang et al., 2022). Data from 1 January 2011, to 31 December 2020, were extracted by a team of respiratory clinicians and data engineers, with rigorous validation through iterative cleaning and cross-referencing against source records. Missing or ambiguous data were resolved via follow-up calls to patients or families, where feasible. The database retrieval study was approved by the Ethics Committee of the West China Hospital, Sichuan University (No. 2020-1056), which waived the requirement for informed consent due to the retrospective nature of the research.

### Study participants

Eligible patients were adults (≥18 years) hospitalized with a primary discharge or death diagnosis of acute exacerbation of COPD (AECOPD), identified using the ICD-10 codes J44.0 (COPD with acute lower respiratory infection) or J44.1 (COPD with acute exacerbation). The diagnosis of COPD was previously confirmed by pulmonologists, with spirometry performed in all of the overall cohort, demonstrating post-bronchodilator FEV1/FVC <0.70 in line with GOLD criteria. The diagnosis of the index AECOPD was further confirmed by pulmonologists through chart review, which required documented acute worsening of COPD-specific symptoms such as dyspnea, increased sputum volume, and purulence per GOLD criteria. Bronchiectasis exacerbations were not considered unless they were concurrent with AECOPD, ensuring that our cohort reflected the true overlap syndrome.

All patients were required to have a chest CT screening for bronchiectasis. Bronchiectasis was identified based on chest CT scans within the 12 months before or during the index hospitalization, meeting at least one of the following radiological criteria: 1) chest CT showing a bronchial diameter greater than the accompanying pulmonary artery diameter (bronchial-to-artery ratio >1); 2) lack of normal tapering of the bronchi; or 3) visualization of bronchi within 1 cm of the pleural surface. The “tram track” sign was considered supportive but not required for diagnosis. All CT scans were reported by certified radiologists and reviewed by the study team.

Exclusion criteria included: 1) coexisting conditions of asthma, cystic fibrosis (CF), acute decompensation of major comorbidities such as heart failure (NYHA Class III or IV), or pulmonary embolism; 2) recent (within 1 month) use of immunosuppressive drugs or biologics; 3) comorbid hematological disorders (e.g., eosinophilia and leukemia); 4) missing critical variables, including eosinophil count, CT results, and in-hospital prognostic outcomes; 5) duration of hospitalization <24 h; 6) medically recorded SCS use for indications other than AECOPD during hospitalization. Asthma involves distinct eosinophilic inflammatory pathways, and its corticosteroids responsiveness is fundamentally different from that of COPD. Including patients with asthma in this study could obscure the interpretation of the role of BEC in COPD-specific exacerbations. CF is a genetic disorder characterized by mucus plugging, chronic *Pseudomonas* infections, and neutrophilic inflammation. Bronchiectasis in CF arises from unique mechanisms, such as CF transmembrane conductance regulator dysfunction, which differs from those in COPD. Therefore, we excluded both documented physician diagnoses of asthma and CF at any time from the analysis to avoid potential confounding factors and ensure an accurate interpretation of the role of eosinophil count in COPD exacerbations.

### Engagement of patients and the public

Given that this study was retrospective in nature, there was no requirement for patient recruitment or the signing of informed consent forms. Furthermore, the patients and public were not involved in the determination of research questions, selection of outcome measures, study design, conduct, or reporting of the research findings.

### Exposure and outcomes

In this study, the core exposure factor was baseline BEC, which was measured within 24 h of admission before any antibiotic or corticosteroids use. To analyze the impact of BEC on modifying clinical outcomes after SCS treatment, it was examined both as a continuous variable and as a categorical variable for further comparison. Based on the different levels of BEC, patients were categorized into two (low BEC <300 cells/μL and high BEC ≥300 cells/μL) or three groups (very low BEC <100 cells/μL, moderate BEC 100 ≤ BEC <300 cells/μL, and high BEC ≥300 cells/μL) (Chalmers and Shoemark, 2023; Global Initiative for Chronic Obstructive Lung Disease, 2024). This categorization followed the relevant criteria outlined in the GOLD guideline and aimed to explore the potential effects of different eosinophil counts on inpatient outcomes in patients with COPD complicated by bronchiectasis. Although these cutoffs have not been formally validated in COPD-bronchiectasis overlap patients, recent evidence from bronchiectasis cohorts supports their biological relevance, providing a plausible framework for application to this population (Martínez-García et al., 2023; Shoemark et al., 2022). We acknowledge that future studies are needed to determine optimal thresholds specific to this overlap syndrome.

The primary outcome was in-hospital mortality (all-cause mortality during hospitalization). Secondary outcomes included: 1) length of hospital stays (LOS); 2) intensive care unit (ICU) admission, 3) infectious complications indicated by the use of antibiotic treatment, anti-fungal treatment, and broad-spectrum antibiotic treatment (BSAT). BSAT was defined as the concurrent use of two or more classes of antibiotics for suspected or confirmed polymicrobial infection, or as empirical therapy for healthcare-associated pneumonia or hospital-acquired pneumonia; 4) non-invasive or invasive mechanical ventilation (NIV/IV) utilization; 5) readmission within 30 days after discharge; and 6) hospitalization expenses.

### Data collection and variable definitions

This study collected detailed demographic and clinical data, including age, sex, body mass index (BMI), and smoking history, from an electronic health platform. We also gathered clinical data, including AECOPD-related hospitalizations in the previous year (AHPY), disease duration, radiological imaging results, and comorbidities, such as gastroesophageal reflux disease, coronary artery disease, hypertension, diabetes, osteoporosis, cerebrovascular accidents, anxiety, and depression.

Clinical data including SCS use, lower respiratory tract infection (LRTI), and antibiotic treatment were also recorded. SCS use during hospitalization was defined as continuous oral or intravenous corticosteroids administration (including dexamethasone, prednisone, methylprednisolone, and others) for at least 3 days. The total dosage of other corticosteroids medications (such as dexamethasone and prednisone) was standardized to methylprednisolone equivalents, based on anti-inflammatory potency ratios. LRTI referred to bacterial or fungal infections, which were confirmed by positive pathogen cultures or smear tests from valid sputum or bronchoalveolar lavage samples collected within the first 24 h of admission, before antibiotic treatment, supported by an increased serum procalcitonin (>0.046 ng/mL) or infectious lesions on CT imaging. Antibiotic treatment was defined as the administration of oral or intravenous antibiotics for at least five consecutive days. The decision to initiate antibiotics was made by the attending pulmonologist based on clinical suspicion of bacterial infection based on clinical symptoms, blood test, pathogen cultures, and/or radiographic infiltrates, following the recommendations of the GOLD guidelines for AECOPD and the guidelines for bronchiectasis exacerbations (Polverino et al., 2017). According to these guidelines, empirical antibiotic selection is stratified by the presence or absence of risk factors for *Pseudomonas aeruginosa* infection.

Data were collected from each patient following the index date, which was defined as the date of hospitalization for AECOPD.

### Bronchiectasis severity assessment

To address the potential influence of bronchiectasis disease burden on outcomes, we performed a post-hoc assessment of severity using available chest CT scans. For all patients in the COPD-bronchiectasis overlap group, the most recent high-resolution CT scan prior to or during the index hospitalization was independently reviewed by two experienced radiologists who were blinded to the clinical outcomes. Discrepancies were resolved by consensus with a third senior pulmonologist. We employed the widely used modified Reiff score for morphological severity assessment (Reiff et al., 1995; Pasteur et al., 2000). This score is based on the number of lung lobes involved, with the lingula considered a separate lobe, resulting in a total of 6 lobes, which ranges from 0 to 18, where the higher the score, the greater the bronchiectasis severity (Mild, score: 1-6; Moderate, score: 7-12, Severe, score: 13-18).

### Statistical analysis

Descriptive statistics were used to summarize the data. For continuous variables, normally distributed data were presented as mean ± standard deviation (Mean ± SD), whereas non-normally distributed data were expressed as medians with interquartile ranges (IQR). For all estimates, 95% confidence interval (CI) was provided where applicable. Comparisons between the two groups were made using the t-test or Mann-Whitney U test for continuous variables, depending on the data distribution, and the chi-square test or Fisher’s exact test was applied for categorical variables. Comparisons between the three groups were made using one-way analysis of variance or Kruskal-Wallis test for continuous variables. The Chi-squared test for homogeneity was used to assess differences in proportions across groups.

Multivariate regression analysis involving binary logistic regression was used to assess the association between eosinophil level and binary outcomes, and linear regression was used to examine the relationship between eosinophil level and continuous outcomes. Different models including different covariates were involved for multivariable regression analysis: Model 1 adjusted for confounders of gender, age, BMI, disease duration, GOLD stage, other comorbidities, smoking status, respiratory failure, AHPY, and existence of LRTI; Model 2 additionally adjusted for SCS use and antibiotic treatment based on Model 1. For the multivariable linear regression analysis examining the relationship between BEC and LOS, a log transformation of LOS was applied if a nonlinear relationship was observed. All reported coefficients for LOS in the results were back-transformed to the original scale to facilitate clinical interpretation. Additionally, Cox proportional hazards models were employed to analyze time-to-event outcomes such as the 30-day readmission rate. To ensure the robustness of the models in linear regression, multicollinearity was tested using the variance inflation factor (VIF), with values <5 considered acceptable. The goodness-of-fit of the models was assessed using the Hosmer-Lemeshow test, with a *P*-value greater than 0.05 indicating a good fit.

The extent and patterns of missing data were assessed for all key variables. To minimize potential bias and retain statistical power, multiple imputations with chained equations were performed to handle missing values in covariates. Five imputed datasets were created, and the imputation model included all variables used in the primary analysis models, as well as the primary outcome. The results from the imputed datasets were pooled using Rubin’s rules.

To address potential confounding and treatment selection bias, propensity score matching (PSM) was performed. For analysis comparing outcomes between SCS-treated and non-SCS-treated patients within the bronchiectasis cohort, patients were matched 1:1 using a caliper width of 0.02 of the standard deviation of the logit of the propensity score, a choice that balances covariate balance and sample size retention in accordance with established methodological recommendations (Austin, 2011). The propensity score was estimated using logistic regression based on covariates including age, sex, BMI, smoking status, disease duration, AHPY, GOLD stage, comorbidities, LRTI, presence of respiratory failure, and antibiotic use. Sensitivity analyses were also conducted using Firth regression to confirm the consistency of the results for rare events.

Subgroup analyses were performed based on factors such as age, BEC categories, and bronchiectasis severity to identify any variations in treatment effects. Interaction terms were tested in multivariable models. Given the exploratory nature of these subgroup analyses, no adjustment for multiple comparisons was applied; results are presented with exact P-values and confidence intervals, and findings are interpreted as hypothesis-generating.

Statistical significance was set at *P* < 0.05, and all analyses were conducted using R version 4.3.1 and SPSS version 26.

### Sample size estimation

Based on previous literature (Ritchie et al., 2023), assuming a 30% reduction in mortality in the high BEC group compared to the control group, α = 0.05, and β = 0.2, the estimated sample size required was at least 500 cases (power = 80%).

## Results

### Characteristics and intergroup comparisons

**FIGURE 1:**
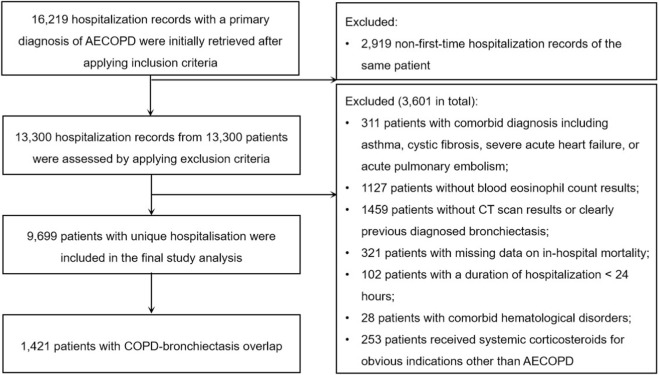
Flow chart of the inclusion process for this cohort study.

After applying the inclusion criteria, 16,219 hospitalization records corresponding to 13,300 unique patients were retrieved. To ensure statistical independence, we included only the first eligible hospitalization for each patient, excluding 2,919 non-first hospitalizations. After applying patient-level exclusion criteria, 3,601 patients were further excluded, which yielded 9,699 patients for analysis. Among them, 1,421 patients (14.65%) had a COPD-bronchiectasis overlap (Figure 1). Compared to COPD-only patients, the bronchiectasis group was younger (71.56 vs. 75.22 years, *P* < 0.001), had a lower proportion of males (*P* < 0.001), longer disease duration (*P* < 0.001), higher rates of type II respiratory failure (*P* < 0.001) and LRTI (*P* < 0.001), and more frequent use of NIV (*P* = 0.017) and BSAT (*P* < 0.001). In-hospital mortality was comparable between groups (*P* = 0.064). Notably, BEC was lower in the overlap group (absolute number: *P* = 0.042; percentage: *P* = 0.031). SCS were more commonly administered to bronchiectasis patients (*P* < 0.001) (Supplementary Table S1). Radiological severity of bronchiectasis was associated with a graded increase in unadjusted in-hospital mortality (*P* = 0.035) (Supplementary Table S2).

The single-variable logistic regression analysis revealed bronchiectasis as associated to an increased risk of in-hospital death in AECOPD patients (OR 1.67, 95% CI 1.31–2.12, *P* < 0.005) (Supplementary Table S3). After multivariable adjustment, the association remained significant (adjusted OR 2.16, 95% CI 1.65–2.82, *P* < 0.005) (Supplementary Table S4), and was confirmed by sensitivity analysis using PSM (OR 2.06, 95% CI, 1.43–2.97; *P* < 0.001). Bronchiectasis was also associated with higher rates of NIV, antibiotic treatment, and the use of double or triple antibiotic therapy (all *P* < 0.05) (Supplementary Table S5). The dependent variable, LOS, was log-transformed due to non-normal distribution, and the reported coefficients were back-transformed to the original scale. Accordingly, the presence of bronchiectasis did not significantly affect LOS (*P* = 0.278; tolerance 0.989, VIF 1.011), potentially reflecting aggressive infection management.

### Factors associated with SCS prescription

Despite traditional concerns that bronchiectasis might discourage SCS use, we found that SCS were prescribed more often to overlap patients with greater disease severity, including longer COPD duration, more frequent prior exacerbations, and type II respiratory failure (Supplementary Table S6). Conversely, a higher BEC was associated with a lower likelihood of SCS administration in both general AECOPD patients (adjusted OR 0.99, *P* < 0.001) and AECOPD-bronchiectasis comorbid patients (all *P* < 0.05) (Supplementary Table S6). In contrast, a higher BEC at admission was associated with a lower likelihood of SCS administration (*P* = 0.001). After adjusting for confounders, the presence of bronchiectasis itself did not independently influence SCS prescription (*P* = 0.254) (Supplementary Table S6). However, a greater objective bronchiectasis severity was indeed related to a lower chance of SCS use in overlap patients (adjusted OR 0.68, *P* = 0.038). These results suggest that disease severity-guided strategies, rather than bronchiectasis diagnosis or eosinophil count alone, critically shape SCS decisions in AECOPD-bronchiectasis comorbidity management in the clinical practice at our hospital.

### Clinical effects of SCS and modulatory role of BEC in AECOPD-bronchiectasis comorbidity

To evaluate the effect of SCS on short-term outcomes in AECOPD patients with bronchiectasis, we performed between-group comparisons. As shown in Table 1, the SCS group exhibited a higher baseline burden of comorbidities or complications, such as chronic pulmonary heart disease (68.11% vs. 58.40%, *P* < 0.001), type II respiratory failure (13.52% vs. 5.02%, *P* < 0.001), and rates of LRTI (77.55% vs. 71.11%, *P* = 0.011). Notably, these patients presented with significantly lower baseline blood eosinophil levels (tested before SCS or antibiotic treatment) (absolute: 153.53 vs. 175.90 cells/μL, *P* = 0.001; percentage: 2.15% vs. 2.66%, *P* < 0.001). Despite more aggressive management, including increased use of NIV and BSAT, SCS administration was associated with a higher in-hospital mortality rate (4.21% vs. 2.20%, OR 1.93, 95% CI 1.01–3.69, *P =* 0.047) and 30-day readmission rate (6.38% vs. 3.30%, OR 2.01, 95% CI 1.21–3.35, *P* = 0.008) in the SCS group, compared with the non-SCS group. Besides, we found that SCS use was much less in patients with moderate and severe bronchiectasis lesions, different from that in patients with mild bronchiectasis (*P* < 0.001) (Table 1).

**TABLE 1:** Comparison of SCS-treated and non-SCS-treated AECOPD-bronchiectasis patients.

Characteristics	SCS use (n = 784)	No SCS use (n = 637)	*P-*value
Age, year, mean (SD)	71.37 ± 10.03	71.79 ± 10.77	0.459
Male, n (%)	522 (66.58)	424 (66.56)	0.994
BMI, mean (SD)	20.52 ± 2.12	20.45 ± 1.96	0.522
Current cigarette smoking, n (%)	61 (7.78)	48 (7.54)	0.863
Disease duration, median (IQR)	10.00 (10.00, 20.00)	10.00 (10.00, 20.00)	<0.001
AHPY, median (IQR)	1.00 (1.00, 2.00)	1.00, (1.00, 2.00)	0.182
GOLD stage, n (%)			
I-II stage	388 (49.49)	410 (64.36)	<0.001
III-IV stage	396 (50.51)	227 (35.64)	
Bronchiectasis severity, n (%)			
Mild	565 (72.07)	379 (59.50)	<0.001
Moderate	151 (19.26)	162 (25.43)	0.005
Severe	68 (8.67)	96 (15.07)	<0.001
Comorbidity, n (%)			
GERD	40 (5.10)	47 (7.38)	0.075
Hypertension	207 (26.40)	188 (29.51)	0.193
CAHD	273 (34.82)	218 (34.22)	0.813
Diabetes	126 (16.07)	116 (18.21)	0.286
Osteoporosis	59 (7.53)	50 (7.85)	0.850
Anxiety	13 (1.66)	6 (0.94)	0.242
Depression	4 (0.51)	6 (0.94)	0.333
Lung cancer	240 (30.61)	205 (32.18)	0.526
CPHD	534 (68.11)	372 (58.40)	<0.001
CPAE	114 (14.54)	91 (14.29)	0.892
Four or more other comorbidities	186 (23.72)	142 (22.29)	0.524
Eosinophils at admission, mean (SD)			
Absolute number, cells/μL	153.53 ± 108.57	175.90 ± 141.52	0.001
Percentage, %	2.15 ± 1.60	2.66 ± 2.02	<0.001
BGA at admission			
PO_2_, median (IQR)	80.50 (70.75, 90.30)	80.50 (78.30, 87.45)	0.108
PCO_2_, median (IQR)	45.80 (45.80, 58.73)	45.80 (42.85, 45.80)	<0.001
Type I respiratory failure, n (%)	185 (23.60)	97 (15.23)	<0.001
Type II respiratory failure, n (%)	106 (13.52)	32 (5.02)	<0.001
LRTI, n (%)	608 (77.55)	453 (71.11)	0.006
Mortality, n (%)	33 (4.21)	14 (2.20)	0.035
Readmission within 30 days, n (%)	50 (6.38)	21 (3.30)	0.008
Ventilator use, n (%)			
NIV	183 (23.34)	59 (9.26)	<0.001
IV	24 (3.04)	13 (2.04)	0.230
LOS, days, median, median (IQR)	12.00 (12.00, 13.00)	12.00 (10.00, 12.00)	<0.001
ICU admission, n (%)	8 (1.02)	3 (0.47)	0.240
Antibiotic treatment, n (%)	781 (99.62)	600 (94.19)	<0.001
BSAT, n (%)	116 (14.80)	50 (7.85)	<0.001
Antifungal treatment, n (%)	86 (10.97)	37 (5.81)	<0.001
Antifungal treatment time, days, median (IQR)	11.00 (7.00, 15.00)	10.00 (7.00, 13.25)	0.590

Abbreviations: BMI, body mass index; SCS, systemic corticosteroids; SD, standard deviation; IQR, interquartile range; AHPY, AECOPD-induced hospitalizations in the past year; BGA, blood gas analysis; NIV, Non-invasive mechanical ventilation; IV, invasive mechanical ventilation; LOS, length of stay; BSAT, Broad-spectrum antibiotic treatment; CPHD, chronic pulmonary heart disease; LRTI, lower respiratory tract infection; GERD, gastroesophageal reflux disease; CAHD, coronary atherosclerotic heart disease; CPAE, chronic pulmonary artery embolism.

However, after multivariable binary logistic regression adjustment for confounders, the association between SCS use and in-hospital mortality was attenuated and no longer statistically significant (OR 1.74, *P* = 0.097) (Supplementary Table S7). Propensity score matching identified 629 well-balanced pairs (Supplementary Figure S1). In this matched cohort, the association between SCS use and in-hospital mortality was further assessed, yielding an adjusted OR of 1.836 (95% CI 0.97–3.46, *P* = 0.060). This suggests that the increased mortality observed in the unadjusted analysis may be largely explained by the greater baseline illness severity in patients selected for SCS treatment, highlighting a significant confounding-by-indication bias. Similarly, the total dosage of SCS was not significantly associated with in-hospital mortality in the adjusted model (B = 0.00, OR 1.00, *P* = 0.115) (Supplementary Table S7). Nevertheless, SCS use remained independently associated with an increased 30-day readmission (*P* = 0.045), utilization of NIV (*P* < 0.001), antibiotic treatment (*P* < 0.001), combined antibiotic treatment (*P* < 0.001), and LOS (*P* = 0.001) (Supplementary Table S8).

We then performed a subgroup analysis to evaluate the modulatory role of BEC. All analyses were conducted within the bronchiectasis-overlap cohort, comparing outcomes between patients who received SCS and those who did not within the same stratification.

Patients were categorized into different subgroups based on BEC levels. As shown in Supplementary Tables S9, S10, no deaths occurred during hospitalization in the high BEC subgroup (BEC ≥300 cells/μL), whereas mortality rates were 4.88% and 4.26% in the very low (<(100 cells/µL) and moderate low (100 ≤ BEC <300 cells/μL) groups, respectively, in the unmatched cohort.

Within the low BEC group (<300 cells/μL), SCS use was associated with significantly higher in-hospital mortality (PSM cohort: SCS group 4.18% [25/597] vs. non-SCS group 2.31% [13/563]; PSM-adjusted OR 2.05, 95% CI 1.04–4.02, *P* = 0.038). While in high BEC group (≥300 cells/μL), SCS use was associated with a reduced need for antibiotic treatment (PSM-adjusted OR 0.71, *P* = 0.022).

**FIGURE 2:**
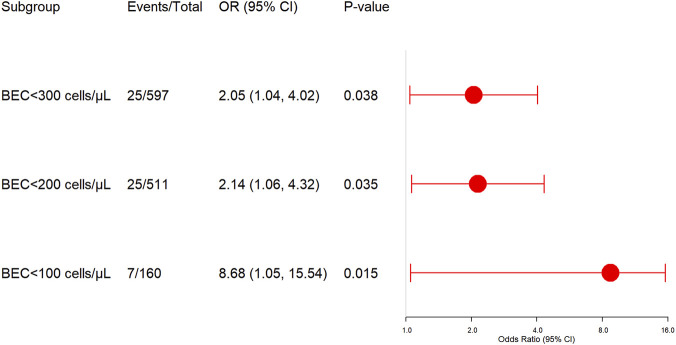
BEC-stratified mortality risk in SCS-treated hospitalized COPD-bronchiectasis patients, compared to their matched non-SCS users in each BEC stratum. OR, Odds ratio; BEC, Blood eosinophil count.

When the low BEC group was further stratified to <200cells/µL and 100 cells/µL, the mortality risk was still pronounced in SCS users compared to non-users (BEC <200cells/µL: PSM cohort: SCS group: 4.89% [25/511] vs. non-SCS group: 2.31% [11/477]; PSM-adjusted OR 2.14, 95% CI 1.06–4.32, *P* = 0.035; BEC <100cells/µL: PSM cohort: SCS group: 4.38% [7/160] vs. non-SCS group: 0.65% [1/153]; PSM-adjusted OR 8.68, 95% CI 1.05–15.54, *P* = 0.015) (Figure 2). To further address the associations between SCS use and mortality, we performed a sensitivity analysis using Firth’s penalized logistic regression for the very low BEC subgroup (<100 cells/μL). The Firth-corrected OR was 4.53 (95% CI 1.01–16.78) (*P* = 0.047), which is more conservative than the original estimate.

**FIGURE 3:**
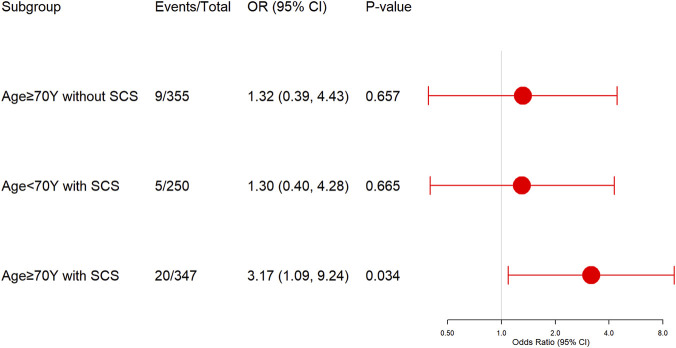
Age-stratified mortality risk associated with SCS use in hospitalized COPD-bronchiectasis patients with BEC <300 cells/μL, compared to the group of age <70 years without SCS use. OR, Odds ratio; SCS, Systemic corticosteroids.

In addition, among patients with BEC <300 cells/μL, a significant interaction was observed between SCS use and age (interaction coefficient B = 0.89, SE = 0.38, OR 2.43, 95% CI 1.15–5.13, interaction *P* = 0.020). In PSM cohort, compared to younger patients (<70 years) not receiving SCS (1.92% [4/208]), elderly patients (≥70 years) who received SCS had a significantly increased risk of in-hospital death (PSM-adjusted OR 3.17, 95% CI 1.09–9.24, *P* = 0.034) (Figure 3).

Then, we examined whether this BEC-dependent risk was influenced by radiological bronchiectasis severity. Among low-BEC patients (<300 cells/µL), the harmful association between SCS use and mortality persisted across all bronchiectasis severity strata but exhibited a graded increase in risk estimates when compared to non-SCS users within the same severity category. The PSM adjusted odds ratio was 1.99 (95% CI 1.01–2.98) (*P* = 0.032) for mild bronchiectasis, 2.15 (95% CI 1.46–3.32) (*P* = 0.047) for moderate bronchiectasis, and 4.56 (95% CI 2.85–5.03) (*P* = 0.024) for severe bronchiectasis. Notably, within the severe bronchiectasis subgroup, the risk associated with SCS was further elevated in patients with very low BEC (<100 cells/μL) (adjusted OR 6.36, 95% CI 4.77–10.54). Furthermore, within this high-risk phenotype (severe bronchiectasis and BEC <100 cells/µL), a strong interaction between SCS and age was observed (interaction coefficient B = 1.32, SE = 0.35, OR 3.74, 95% CI 1.88–7.44, *P* < 0.001). Elderly patients (≥70 years) receiving SCS had a 3.28-fold higher mortality risk (adjusted OR 3.28, 95% CI 1.30–6.12, *P* < 0.001) compared to younger patients (<70 years) within the same treatment and disease-severity subgroup.

We also evaluated the impact of eosinophils on secondary outcomes in patients with general COPD-bronchiectasis overlap with SCS-treated patients. Cox analysis showed that an increased eosinophil count indicated a reduced risk of readmission within 30 days (OR 0.99, 95% CI 0.99–1.00; *P* = 0.025) (Supplementary Table S11), and linear regression analysis showed that it was also associated with a shorter LOS (R^2^ = 0.30, *P* = 0.016) (Supplementary Table S12). No significant association was observed between BEC and other secondary outcomes in this population after adjustment (Supplementary Table S13).

## Discussion

This retrospective cohort study examined the management and outcomes of SCS in hospitalized patients with AECOPD and comorbid bronchiectasis. Our analysis suggested SCS was more frequently administered to patients with greater objective disease severity, who paradoxically tended to have lower baseline BEC. This pattern introduces two distinct but interrelated methodological challenges that complicate causal inference (Global Initiative for Chronic Obstructive Lung Disease, 2024). The first challenge, confounding by indication, arises because clinicians preferentially prescribed SCS to patients with more severe clinical presentations, those with longer disease duration, higher rates of type II respiratory failure, and greater infection burden. The higher unadjusted mortality in the SCS group likely reflects baseline illness severity rather than SCS toxicity, supported by attenuation after multivariable adjustment. The second challenge involves reverse causality. Low BEC at admission may itself indicate acute physiological stress, sepsis, or a pronounced systemic inflammatory response (Tashkin and Wechsler, 2018), all independently associated with worse outcomes. In critically ill patients, eosinopenia is a well-recognized stress response driven by endogenous cortisol release and inflammatory cytokine-mediated suppression of eosinophil production and survival. In AECOPD with bronchiectasis, a population characterized by chronic bacterial colonization and recurrent infections, low BEC may thus identify patients with active infection, heightened systemic inflammation, or impending sepsis. This creates a fundamental interpretive dilemma: if low BEC serves as a marker of underlying disease severity rather than a pure modifier of corticosteroid responsiveness, the observed association between SCS use and mortality in this subgroup may partly reflect higher intrinsic risk rather than a direct harmful effect of treatment. Hence, the causal pathway is bidirectional, patients with low BEC are more likely to receive SCS due to perceived severity and more likely to have adverse outcomes due to their underlying condition, making it difficult to disentangle the independent contribution of SCS from baseline risk. This practice persists despite evidence that low BEC exacerbations are often infection-driven and may derive limited benefit, or even harm, from immunosuppressive therapy (Global Initiative for Chronic Obstructive Lung Disease, 2024; Prins et al., 2017; Donnan et al., 2025; Jabarkhil et al., 2020). Collectively, this “severity-response bias” may explain the unadjusted association between SCS use and increased mortality in low-BEC patients. If confirmed in future studies, these findings would underscore the need to move beyond a syndromic approach toward a “treatable traits” framework, where therapeutic decisions are guided by underlying inflammatory endotypes rather than diagnostic labels or clinical severity alone (David et al., 2021).

Our findings, while hypothesis-generating, suggest a potential clinically relevant risk-stratification framework that warrants further evaluation. Specifically, SCS use in low-BEC patients, particularly those aged ≥70 years, was associated with increased mortality. This may reflect that low BEC often indicates an infection-driven pathology where SCS offers limited anti-inflammatory benefit while raising infection risk, especially in the context of chronic airway colonization and age-related susceptibility to SCS adverse effects (Battaglia et al., 2014; Ma et al., 2024). Critically, BEC <100 cells/μL and age ≥70 years act as synergistic risk factors, and the risk gradient intensifies with radiological bronchiectasis severity. Thus, the highest-risk phenotype is an elderly patient (≥70 years) with very low BEC (<100 cells/μL) and severe bronchiectasis. In contrast, SCS use in patients with BEC ≥300 cells/μL was not associated with excess mortality and was linked to reduced antibiotic need, supporting eosinophils as a biomarker of Th2 inflammation responsive to corticosteroids. If prospectively validated, these observations could translate into actionable clinical insights. For patients with BEC ≥300 cells/μL, SCS may be used without added mortality concern and may lower antibiotic requirements. For those with BEC <300 cells/μL and age ≥70 years, a cautious, infection-focused strategy should be prioritized, avoiding routine SCS.

Recent trials (CORTICO-COP and STARR2) reported no short-term mortality or LOS benefit from eosinophil-guided SCS reduction in general COPD exacerbations (Sivapalan et al., 2019; Ramakrishnan et al., 2024), they did not address COPD-bronchiectasis overlap. While studies in bronchietasis or chronic bronchial infection overlapped COPD populations demonstrated BEC-dependent modification of pneumonia and exacerbation risk (Ritchie et al., 2023; Martinez-Garcia et al., 2020), but none examined SCS use in relation to mortality. Our study therefore provides preliminary, hypothesis-generating evidence that BEC modifies the association between SCS and in-hospital mortality in COPD-bronchiectasis overlap, identifying a high-risk subgroup where SCS may be harmful. This finding underscores the importance of phenotype-guided SCS decisions during acute exacerbations, a dimension overlooked in current RCTs.

This study has several important limitations inherent to its observational design and methodological constraints. First, despite employing multivariable adjustment and PSM, residual confounding from the unmeasured variables may persist. Key indicators of acute illness severity, such as C-reactive protein, lactate, components of the Sequential Organ Failure Assessment score, hemodynamic parameters, and immunosuppression indicators, were not available in our database. Clinicians may have preferentially prescribed systemic corticosteroids to patients with greater physiological derangement, and such unmeasured severity markers could contribute to residual confounding. Second, the single measurement of BEC at admission may not capture dynamic changes during hospitalization or reflect prior exacerbation phenotypes (Donnan et al., 2025; Kang et al., 2016). This is particularly relevant given that low BEC can also represent acute stress responses or sepsis-related eosinopenia, introducing potential reverse causality where BEC serves as a marker of illness severity rather than a genuine effect modifier. Third, the clinical applicability of the eosinophil count threshold requires further validation. Although the ≥300 cells/μL cutoff aligns with existing COPD literature, it has not been validated specifically for guiding systemic corticosteroid use in hospitalized patients with bronchiectasis overlap. Future RCTs must validate BEC-stratified protocols to translate these hypothesis-generating findings into clinical practice. Fourth, heterogeneity in disease classification may have introduced bias. Although bronchiectasis was radiologically confirmed, the absence of standardized severity scoring such as Reiff score in the primary analysis may have introduced heterogeneity in disease classification (Reiff et al., 1995). Additionally, while we excluded patients with overt non-COPD causes of acute deterioration, subclinical cardiac dysfunction may have contributed to symptom exacerbation in elderly patients with COPD. Future studies should integrate validated severity scales to better stratify risk within this heterogeneous population. Fifth, generalizability and treatment heterogeneity limit the interpretation of findings. This single-center tertiary cohort from China may not reflect global practices or ethnic and genetic variations treatment responses. Therapeutic heterogeneity including antibiotic protocols and SCS tapering practices remained unstandardized over the decade-long study period, potentially confounding outcome interpretation. Moreover, we retained only the first hospitalization per patient to avoid statistical bias from repeated measures; while this strengthens the validity of our associations, it may not fully capture the long-term treatment patterns and outcomes for patients with frequently exacerbating disease. Future studies could consider adopting longitudinal data analysis methods to account for all exacerbation events. Sixth, the exploratory nature of our subgroup analyses involved multiple hypothesis tests, which increases the risk of type I error. We did not adjust for multiplicity because the analyses were intended to generate hypotheses rather than provide confirmatory evidence. Consequently, the findings from subgroup analyses should be interpreted cautiously and validated in independent prospective cohorts. Moreover, after PSM, the sample sizes in certain subgroups, such as the very low BEC stratum, became relatively small, which may lead to unstable effect estimates and warrants cautious interpretation.

## Conclusion

In summary, this study suggests that blood eosinophil count may serve as a useful biomarker to guide the use of SCS in hospitalized patients with COPD-bronchiectasis overlap, though this requires prospective validation. Particular caution may be warranted in a high-risk phenotype characterized by elderly patients (≥70 years) with very low BEC (<100 cells/μL), especially when accompanied by severe radiological bronchiectasis, as the risks of SCS therapy could outweigh its benefits in this subgroup. These findings, while hypothesis-generating, support the adoption of a phenotype-driven treatment strategy for further investigation and underscore the need for prospective randomized trials to validate the role of BEC in optimizing management for this complex patient population.

## Data Availability

The original contributions presented in the study are included in the article/Supplementary Material, further inquiries can be directed to the corresponding authors.

## Supplementary material

SUPPLEMENTARY FIGURE S1 Love plot showing standardized mean differences before and after propensity score matching. BMI, Body mass index; AHPY, AECOPD-induced hospitalizations in the past year; LRTI, Lower respiratory tract infection.

## References

[B1] Austin P. C. (2011). Optimal caliper widths for propensity-score matching when estimating differences in means and differences in proportions in observational studies. Pharm. Statistics 10 (2), 150–161. 10.1002/pst.433 20925139 PMC3120982

[B2] Battaglia S. Cardillo I. Lavorini F. Spatafora M. Scichilone N. (2014). Safety considerations of inhaled corticosteroids in the elderly. Drugs & Aging 31 (11), 787–796. 10.1007/s40266-014-0213-1 25212953

[B3] Bhatt S. P. Rabe K. F. Hanania N. A. Vogelmeier C. F. Cole J. Bafadhel M. (2023). Dupilumab for COPD with type 2 inflammation indicated by eosinophil counts. N. Engl. Journal Medicine 389 (3), 205–214. 10.1056/NEJMoa2303951 37272521

[B4] Chalmers J. D. Shoemark A. (2023). Inhaled corticosteroids in COPD and bronchiectasis: use biomarkers rather than disease labels. Chest 164 (4), 809–811. 10.1016/j.chest.2023.07.013 37805235

[B5] David B. Bafadhel M. Koenderman L. De Soyza A. (2021). Eosinophilic inflammation in COPD: from an inflammatory marker to a treatable trait. Thorax 76 (2), 188–195. 10.1136/thoraxjnl-2020-215167 33122447 PMC7815887

[B6] Donnan M. Liu T. L. Gvalda M. Chen X. Foo C. T. MacDonald M. I. (2025). Clinical characteristics and outcomes of eosinophilic exacerbations of COPD. Int. J. Chron. Obstruct Pulmon Dis. 20, 1061–1070. 10.2147/COPD.S485246 40247922 PMC12005209

[B7] Everaerts S. Lagrou K. Vermeersch K. Dupont L. J. Vanaudenaerde B. M. Janssens W. (2018). Aspergillus fumigatus detection and risk factors in patients with COPD-bronchiectasis overlap. Int. Journal Molecular Sciences 19 (2), 523. 10.3390/ijms19020523 29425123 PMC5855745

[B8] Global Initiative for Chronic Obstructive Lung Disease (2024). Global strategy for the diagnosis, management, and prevention of chronic obstructive pulmonary disease.

[B9] Goeminne P. C. Nawrot T. S. Ruttens D. Seys S. Dupont L. J. (2014). Mortality in non-cystic fibrosis bronchiectasis: a prospective cohort analysis. Respir. Medicine 108 (2), 287–296. 10.1016/j.rmed.2013.12.015 24445062

[B10] Hurst J. R. Elborn J. S. De Soyza A. , and BRONCH-UK Consortium (2015). COPD-bronchiectasis overlap syndrome. Eur. Respir. J. 45 (2), 310–313. 10.1183/09031936.00170014 25653262

[B11] Jabarkhil A. Moberg M. Janner J. Petersen M. N. Jensen C. B. Henrik Äangquist L. (2020). Elevated blood eosinophils in acute COPD exacerbations: better short- and long-term prognosis. Eur. Clinical Respiratory Journal 7 (1), 1757274. 10.1080/20018525.2020.1757274 32489532 PMC7241534

[B12] Kang H. S. Rhee C. K. Kim S. K. Kim J. W. Lee S. H. Yoon H. K. (2016). Comparison of the clinical characteristics and treatment outcomes of patients requiring hospital admission to treat eosinophilic and neutrophilic exacerbations of COPD. Int. J. Chron. Obstruct Pulmon Dis. 11, 2467–2473. 10.2147/COPD.S116072 27757029 PMC5055104

[B13] Ma J. Liu Y. Sun Y. Guo C. Yang G. (2024). Increased pneumonia risk associated with concomitant use of inhaled corticosteroids and benzodiazepines: a pharmacovigilance analysis. Lung 202 (5), 673–681. 10.1007/s00408-024-00741-y 39191908

[B14] Martínez-García M. A. de la Rosa Carrillo D. Soler-Cataluña J. J. Donat-Sanz Y. Serra P. C. Lerma M. A. (2013). Prognostic value of bronchiectasis in patients with moderate-to-severe chronic obstructive pulmonary disease. Am. J. Respir. Crit. Care Med. 187 (8), 823–831. 10.1164/rccm.201208-1518OC 23392438

[B15] Martinez-Garcia M. A. Faner R. Oscullo G. de la Rosa D. Soler-Cataluña J. J. Ballester M. (2020). Inhaled steroids, circulating eosinophils, chronic airway infection, and pneumonia risk in chronic obstructive pulmonary disease. A network analysis. Am. J. Respir. Crit. Care Med. 201 (9), 1078–1085. 10.1164/rccm.201908-1550OC 31922913

[B16] Martínez-García M. Méndez R. Olveira C. Girón R. García-Clemente M. Máiz L. (2023). The U-shaped relationship between eosinophil count and bronchiectasis severity: the effect of inhaled corticosteroids. Chest 164 (3), 606–613. 10.1016/j.chest.2023.04.029 37088355

[B17] Ni Y. Shi G. Yu Y. Hao J. Chen T. Song H. (2015). Clinical characteristics of patients with chronic obstructive pulmonary disease with comorbid bronchiectasis: a systemic review and meta-analysis. Int. J. Chron. Obstruct Pulmon Dis. 10, 1465–1475. 10.2147/COPD.S83910 26251586 PMC4524532

[B18] Pasteur M. C. Helliwell S. M. Houghton S. J. Webb S. C. Foweraker J. E. Coulden R. A. (2000). An investigation into causative factors in patients with bronchiectasis. Am. J. Respir. Crit. Care Med. 162 (4 Pt 1), 1277–1284. 10.1164/ajrccm.162.4.9906120 11029331

[B19] Polverino E. Goeminne P. C. McDonnell M. J. Aliberti S. Marshall S. E. Loebinger M. R. (2017). European respiratory society guidelines for the management of adult bronchiectasis. Eur. Respir. J. 50 (3), 1700629. 10.1183/13993003.00629-2017 28889110

[B20] Polverino E. Dimakou K. Hurst J. Martinez-Garcia M. A. Miravitlles M. Paggiaro P. (2018). The overlap between bronchiectasis and chronic airway diseases: state of the art and future directions. Eur. Respir. J. 52 (3), 1800328. 10.1183/13993003.00328-2018 30049739

[B21] Prins H. J. Duijkers R. Lutter R. Daniels J. M. van der Valk P. Schoorl M. (2017). Blood eosinophilia as a marker of early and late treatment failure in severe acute exacerbations of COPD. Respir. Medicine 131, 118–124. 10.1016/j.rmed.2017.07.064 28947018

[B22] Ramakrishnan S. Jeffers H. Langford-Wiley B. Davies J. Thulborn S. J. Mahdi M. (2024). Blood eosinophil-guided oral prednisolone for COPD exacerbations in primary care in the UK (STARR2): a non-inferiority, multicentre, double-blind, placebo-controlled, randomised controlled trial. Lancet Respir. Medicine 12 (1), 67–77. 10.1016/S2213-2600(23)00298-9 37924830

[B23] Reiff D. B. Wells A. U. Carr D. H. Cole P. J. Hansell D. M. (1995). CT findings in bronchiectasis: limited value in distinguishing between idiopathic and specific types. AJR Am. Journal Roentgenology 165 (2), 261–267. 10.2214/ajr.165.2.7618537 7618537

[B24] Ritchie A. I. Singayagam A. Mitchell S. Wedzicha J. A. Shah A. Bloom C. I. (2023). The effect of inhaled corticosteroids on pneumonia risk in patients with COPD-bronchiectasis overlap: a UK population-based case-control study. Chest 164 (4), 875–884. 10.1016/j.chest.2023.06.007 37419145 PMC10808068

[B25] Shoemark A. Shteinberg M. De Soyza A. Haworth C. S. Richardson H. Gao Y. (2022). Characterization of eosinophilic bronchiectasis: a European multicohort study. Am. J. Respir. Crit. Care Med. 205 (8), 894–902. 10.1164/rccm.202108-1889OC 35050830

[B26] Sivapalan P. Lapperre T. S. Janner J. Laub R. R. Moberg M. Bech C. S. (2019). Eosinophil-guided corticosteroid therapy in patients admitted to hospital with COPD exacerbation (CORTICO-COP): a multicentre, randomised, controlled, open-label, non-inferiority trial. Lancet Respir. Medicine 7 (8), 699–709. 10.1016/S2213-2600(19)30176-6 31122894

[B27] Tashkin D. P. Wechsler M. E. (2018). Role of eosinophils in airway inflammation of chronic obstructive pulmonary disease. Int. J. Chron. Obstruct Pulmon Dis. 13, 335–349. 10.2147/COPD.S152291 29403271 PMC5777380

[B28] Yang M. Liu X. Hu Q. Li J. Fu S. Chen D. (2022). Eosinopenia as a biomarker for antibiotic use in COPD exacerbations: protocol for a retrospective hospital-based cohort study. BMJ Open 12 (1), e051939. 10.1136/bmjopen-2021-051939 35058259 PMC8783821

