# Supplementary material for: Association between blood eosinophil count and in-hospital mortality among systemic corticosteroids-treated patients with COPD-bronchiectasis overlap: a retrospective cohort study
Source: Front Pharmacol. 2026 Apr 29;17:1781776. doi: 10.3389/fphar.2026.1781776 (PMC13167571; doi:10.3389/fphar.2026.1781776)
Supplement: Supplementary file 1 [file Supplementaryfile1.docx]

**Table S1 Baseline characteristics and outcomes of hospitalized AECOPD patients with vs. without bronchiectasis**

| **Characteristics** | **Total**  **(n = 9,699)** | **With bronchiectasis**  **(n = 1,421)** | **Without bronchiectasis**  **(n = 8,278)** | ***P*-value ^*^** |
| --- | --- | --- | --- | --- |
| Age, year, mean (SD) | 74.68±10.59 | 71.56±10.37 | 75.22±10.54 | <0.001 |
| Male, n (%) | 6982 (71.99) | 946 (66.57) | 6036 (72.92) | <0.001 |
| BMI, mean (SD) | 20.57±2.28 | 20.49±2.05 | 20.58±2.32 | 0.355 |
| Current cigarette smoking, n (%) | 715 (7.37) | 109 (7.67) | 605 (7.31) | 0.629 |
| Disease duration, median (IQR) | 10.00 (9.00, 20.00) | 10.00 (10.00, 20.00) | 10.00 (8.00, 20.00) | <0.001 |
| AHPY, median (IQR) | 1.00 (1.00, 2.00) | 1.00 (1.00, 2.00) | 1.00 (1.00, 2.00) | 0.709 |
| **GOLD stage, n (%)** | | | | |
| I-II stage | 3,689 (38.03) | 851 (59.89) | 2,838 (34.28) | <0.001 |
| III-IV stage | 6,010 (61.97) | 570 (40.11) | 5,440 (65.72) |  |
| **Comorbidity, n (%)** | | | | |
| GERD | 600 (6.19) | 87 (6.12) | 513 (6.20) | 0.914 |
| Hypertension | 3818 (39.36) | 395 (27.80) | 3423 (41.35) | <0.001 |
| CAHD | 3591 (37.02) | 491 (34.55) | 3100 (37.45) | 0.037 |
| Diabetes | 2016 (20.79) | 242 (17.03) | 1774 (21.43) | <0.001 |
| Osteoporosis | 767 (7.91) | 109 (7.67) | 658 (7.95) | 0.720 |
| Anxiety | 123 (1.27) | 19 (1.34) | 104 (1.30) | 0.802 |
| Depression | 142 (1.46) | 10 (0.70) | 132 (1.60) | 0.010 |
| Lung cancer | 2882 (29.71) | 445 (31.32) | 2437 (29.44) | 0.153 |
| CPHD | 5505 (56.76) | 906 (63.76) | 4599 (55.56) | <0.001 |
| CPAE | 1021 (10.53) | 205 (14.43) | 816 (9.86) | <0.001 |
| Four or more comorbidities | 2167 (22.34) | 328 (23.03) | 1839 (22.22) | 0.494 |
| **Eosinophils at admission, mean (SD)** | | | | |
| Absolute number, cells/μL | 174.33±215.81 | 163.56±124.88 | 176.18±227.76 | 0.042 |
| Percentage, % | 2.50±2.26 | 2.38±1.82 | 2.52±2.32 | 0.031 |
| **BGA at admission** | | | | |
| PO2, median (IQR) | 80.50 (76.00, 86.50) | 80.50 (73.95, 89.75) | 80.50 (76.40, 85.90) | >0.999 |
| PCO2, median (IQR) | 45.80 (44.00, 47.90) | 45.80 (44.85, 53.00) | 45.80 (43.80, 47.10) | <0.001 |
| Type I respiratory failure, n (%) | 1769 (18.24) | 282 (19.85) | 1487 (17.96) | 0.090 |
| Type II respiratory failure, n (%) | 686 (7.07) | 138 (9.71) | 548 (6.62) | <0.001 |
| LRTI at admission, n (%) | 6397 (65.96) | 1061 (74.67) | 5336 (64.46) | <0.001 |
| Mortality, n (%) | 416 (4.29) | 48 (3.38) | 369 (4.46) | 0.064 |
| Readmission within 30 days, n (%) | 503 (5.19) | 71 (5.00) | 432 (5.22) | 0.727 |
| **Ventilator use, n (%)** | | | | |
| NIV | 1449 (14.94) | 242 (17.03) | 1207 (14.58) | 0.017 |
| IV | 306 (3.15) | 37 (2.60) | 269 (3.25) | 0.198 |
| LOS, days, median, median (IQR) | 12.00 (11.00, 13.00) | 12.00 (11.00, 12.00) | 12.00 (11.00, 13.00) | 0.030 |
| ICU admission, n (%) | 105 (1.08) | 11 (0.77) | 94 (1.14) | 0.224 |
| **Medical treatment** | | | | |
| Antibiotic treatment, n (%) | 9090 (93.72) | 1381 (97.19) | 7709 (93.13) | <0.001 |
| BSAT, n (%) | 853 (8.79) | 166 (11.68) | 687 (8.30) | <0.001 |
| Antifungal treatment, n (%) | 850 (8.76) | 123 (8.66) | 727 (8.78) | 0.876 |
| Antifungal treatment time, days, median (IQR) | 10.00 (6.00, 15.00) | 11.00 (7.00,15.00) | 10.00 (6.00, 15.00) | 0.680 |
| SCS treatment, n (%) | 4944 (50.97) | 784 (55.17) | 4160 (50.25) | <0.001 |
| SCS treatment time, days, median (IQR) | 8.00 (5.00, 12.00) | 9.00 (6.00, 13.00) | 8.00 (5.00, 12.00) | 0.004 |
| SCS treatment total dose, mg, median (IQR) ‡ | 280.00 (160.00, 390.00) | 300.00 (200.00, 400.00) | 280.00 (160.00, 390.00) | 0.016 |

**Notes:**

^*^ AECOPD-bronchiectasis overlap patients vs. AECOPD only patients.

^‡^ SCS doses were standardized to methylprednisolone equivalents based on anti-inflammatory potency ratios.

**Abbreviations:** BMI, Body mass index; SD, Standard deviation; IQR, Interquartile range; AHPY, AECOPD-induced hospitalization in the past year; BGA, Blood gas analysis; NIV, Non-invasive mechanical ventilation; IV, Invasive mechanical ventilation; LOS, Length of stay; BSAT, Broad-spectrum antibiotic treatment; CPHD, Chronic pulmonary heart disease; LRTI, Lower respiratory tract infection; GERD, Gastroesophageal reflux disease; CAHD, Coronary atherosclerotic heart disease; CPAE, Chronic pulmonary artery embolism; ICU, intensive care unit; SCS, Systemic corticosteroids.

**Table S2 Baseline characteristics stratified by bronchiectasis severity grade in AECOPD patients**

| **Characteristics** | **Bronchiectasis-COPD overlap patients (n = 1,421)** | | | ***P*-value** |
| --- | --- | --- | --- | --- |
|  | **Mild (n = 944)** | **Moderate (n = 313)** | **Severe (n = 164)** |  |
| Age, year, mean (SD) | 70.32±9.44 | 72.41±10.07 | 73.25±10.18 | <0.001 |
| Male, n (%) | 618 (65.47) | 221 (70.61) | 107 (65.24) | 0.230 |
| BMI, mean (SD) | 20.55±2.22 | 20.17±2.45 | 20.05±2.14 | 0.004 |
| Current cigarette smoking, n (%) | 65 (6.89) | 29 (9.27) | 15 (9.15) | 0.439 |
| Disease duration, median (IQR) | 10.00 (10.00, 20.00) | 10.00 (11.00, 20.00) | 10.00 (11.00, 20.00) | 0.687 |
| AHPY, median (IQR) | 1.00 (1.00, 2.00) | 1.00 (1.00, 2.00) | 1.00 (1.00, 2.00) | 0.559 |
| **GOLD stage, n (%)** | | | | |
| I-II stage | 533 (56.46) | 220 (70.29) | 98 (59.76) | <0.001 |
| III-IV stage | 411 (43.54) | 93 (29.71) | 66 (40.24) |  |
| **Comorbidity, n (%)** | | | | |
| GERD | 49 (5.19) | 25 (7.99) | 13 (7.93) | 0.120 |
| Hypertension | 95 (10.06) | 199 (63.58) | 101 (61.59) | <0.001 |
| CAHD | 288 (30.51) | 142 (45.37) | 61 (37.20) | <0.001 |
| Diabetes | 147 (15.57) | 69 (22.04) | 36 (15.85) | 0.011 |
| Osteoporosis | 71 (7.52) | 25 (7.99) | 13 (7.93) | 0.956 |
| Anxiety | 10 (1.06) | 5 (1.60) | 4 (2.44) | 0.329 |
| Depression | 5 (0.53) | 3 (0.96) | 2 (1.22) | 0.516 |
| Lung cancer | 263 (27.86) | 120 (38.34) | 62 (37.80) | <0.001 |
| CPHD | 571 (60.49) | 213 (68.05) | 142 (86.59) | <0.001 |
| CPAE | 140 (14.83) | 39 (12.46) | 26 (15.85) | 0.503 |
| Four or more comorbidities | 201 (21.29) | 75 (23.96) | 52 (31.71) | 0.013 |
| **Eosinophils at admission, mean (SD)** | | | | |
| Absolute number, cells/μL | 193.50±117.52 | 161.47±125.43 | 126.58±120.28 | <0.001 |
| Percentage, % | 2.59±2.45 | 2.47±1.55 | 2.00±1.77 | 0.007 |
| **BGA at admission** | | | | |
| PO_2_, median (IQR) | 80.47 (75.57, 88.48) | 80.00 (72.52, 87.68) | 72.54 (70.28, 81.47) | 0.993 |
| PCO_2_, median (IQR) | 43.65 (44.14, 50.37) | 45.67 (44.67, 52.75) | 49.55 (45.80, 58.63) | 0.969 |
| Type I respiratory failure, n (%) | 187 (19.81) | 55 (17.57) | 40 (24.39) | 0.207 |
| Type II respiratory failure, n (%) | 57 (6.04) | 48 (15.34) | 33 (20.12) | <0.001 |
| LRTI at admission, n (%) | 676 (71.92) | 220 (70.29) | 162 (98.78) | <0.001 |
| Mortality, n (%) | 26 (2.75) | 11 (3.51) | 11 (6.71) | 0.035 |
| Readmission within 30 days, n (%) | 45 (4.77) | 11 (3.51) | 15 (9.15) | 0.024 |
| **Ventilator use, n (%)** | | | | |
| NIV | 118 (12.50) | 67 (21.41) | 57 (34.86) | <0.001 |
| IV | 21 (2.22) | 8 (2.56) | 8 (4.88) | 0.143 |
| LOS, days, median, median (IQR) | 12.00 (10.00, 12.00) | 12.00 (11.00, 12.00) | 12.00 (11.00, 13.00) | 0.066 |
| ICU admission, n (%) | 5 (0.53) | 2 (0.64) | 4 (2.44) | 0.035 |
| **Medical treatment** | | | | |
| Antibiotic treatment, n (%) | 912 (96.61) | 305 (97.44) | 164 (100) | 0.051 |
| BSAT, n (%) | 75 (7.94) | 48 (15.34) | 43 (26.22) | <0.001 |
| Antifungal treatment, n (%) | 52 (5.51) | 40 (12.78) | 31 (18.90) | <0.001 |
| Antifungal treatment time, days | 9.00 (6.00, 14.00) | 10.00 (7.00,15.00) | 12.00 (8.00, 17.00) | 0.920 |
| SCS treatment, n (%) | 565 (59.85) | 151 (48.24) | 68 (41.46) | <0.001 |
| SCS treatment time, days, median (IQR) | 8.00 (5.00, 12.00) | 9.00 (6.00, 13.00) | 8.00 (5.00, 12.00) | 0.991 |
| SCS treatment total dose, mg, median (IQR) ^*^ | 300.00 (150.00, 380.00) | 280.00 (180.00, 390.00) | 280.00 (160.00, 380.00) | 0.986 |

**Notes:**

^*^ SCS doses were standardized to methylprednisolone equivalents based on anti-inflammatory potency ratios.

**Abbreviations:** BMI, Body mass index; SCS, Systemic corticosteroids; SD, Standard deviation; IQR, Interquartile range; AHPY, AECOPD-induced hospitalizations in the past year; BGA, Blood gas analysis; NIV, Non-invasive mechanical ventilation; IV, Invasive mechanical ventilation; LOS, Length of stay; BSAT, Broad-spectrum antibiotic treatment; GOLD, Global Initiative for Chronic Obstructive Lung Disease; CPHD, Chronic pulmonary heart disease; LRTI, Lower respiratory tract infection; GERD, Gastroesophageal reflux disease; CAHD, Coronary atherosclerotic heart disease; CPAE, Chronic pulmonary artery embolism.

**Table S3 Impact of bronchiectasis on outcomes in AECOPD patients**

| **Outcomes** | **Unadjusted OR (95% CI)** | ***P*-value** | **Adjusted OR (95% CI)** | ***P*-value** |
| --- | --- | --- | --- | --- |
| In-hospital mortality * | 1.67 (1.31, 2.12) | <0.001 | 2.16 (1.65, 2.82) | <0.001 |
| Readmission within 30 days * | 0.96 (0.74, 1.24) | 0.727 | 1.00 (0.76, 1.33) | 0.976 |
| NIV * | 1.20 (1.03, 1.40) | 0.017 | 1.21 (1.02, 1.43) | 0.032 |
| IV * | 0.80 (0.56, 1.13) | 0.199 | 0.87 (0.60, 1.26) | 0.464 |
| ICU admission * | 0.68 (0.36, 1.27) | 0.227 | 0.69 (0.36, 1.34) | 0.273 |
| Antibiotic treatment ‡ | 2.55 (1.84, 3.53) | <0.001 | 2.25 (1.61, 3.16) | <0.001 |
| BSAT ‡ | 1.46 (1.22, 1.75) | <0.001 | 1.56 (1.29, 1.90) | <0.001 |

**Notes:**

^*^ Adjusted for covariates in Model 2.

^‡^ Adjusted for covariates in Model 1.

**Abbreviations:** ICU, intensive care unit; NIV, Non-invasive mechanical ventilation; IV, Invasive mechanical ventilation; BSAT, Broader-spectrum of antibiotic treatment; OR, Odds ratio.

**Table S4 Multivariable logistic regression analysis for the impact of covariates on in-hospital mortality in AECOPD patients**

| **Model** | **Covariates** | **B** | **SD** | **OR** | **95% CI** | | ***P*-value** | **Hosmer-Lemeshow *P*-value** |
| --- | --- | --- | --- | --- | --- | --- | --- | --- |
|  |  |  |  |  | **Lower Limit** | **Upper Limit** |  |  |
| **1** | Bronchiectasis | 0.76 | 0.14 | 2.15 | 1.64 | 2.81 | <0.001 | 0.652 |
|  | Disease duration | -0.01 | 0.01 | 0.99 | 0.98 | 1.01 | 0.287 |  |
|  | Gender (Male) | 0.11 | 0.13 | 1.11 | 0.86 | 1.43 | 0.407 |  |
|  | Age | 0.01 | 0.01 | 1.01 | 1.00 | 1.02 | 0.152 |  |
|  | BMI | 0.01 | 0.02 | 1.01 | 0.96 | 1.06 | 0.719 |  |
|  | Current cigarette smoking | -0.03 | 0.22 | 0.97 | 0.63 | 1.49 | 0.877 |  |
|  | GOLD stage | 0.22 | 0.35 | 1.30 | 0.99 | 1.65 | 0.098 |  |
|  | Comorbidity ^*^ | 0.09 | 0.14 | 1.09 | 0.83 | 1.45 | 0.532 |  |
|  | AHPY | -0.03 | 0.06 | 0.97 | 0.87 | 1.09 | 0.621 |  |
|  | Type II respiratory failure | 0.23 | 0.20 | 1.26 | 0.85 | 1.86 | 0.259 |  |
|  | LRTI | 0.08 | 0.12 | 1.08 | 0.85 | 1.37 | 0.516 |  |
| **2** | Bronchiectasis | 0.77 | 0.13 | 2.16 | 1.65 | 2.82 | <0.001 | 0.656 |
|  | Disease duration | -0.01 | 0.01 | 1.00 | 0.98 | 1.01 | 0.311 |  |
|  | Gender (Male) | 0.11 | 0.11 | 1.11 | 0.87 | 1.43 | 0.404 |  |
|  | Age | 0.01 | 0.01 | 1.01 | 1.00 | 1.02 | 0.165 |  |
|  | BMI | 0.01 | 0.02 | 1.01 | 0.96 | 1.06 | 0.715 |  |
|  | Current cigarette smoking | -0.03 | 0.20 | 0.97 | 0.63 | 1.49 | 0.887 |  |
|  | GOLD stage | 0.21 | 0.33 | 1.21 | 0.98 | 1.62 | 0.096 |  |
|  | Comorbidity ^*^ | 0.09 | 0.13 | 1.09 | 0.83 | 1.45 | 0.536 |  |
|  | AHPY | -0.03 | 0.05 | 0.97 | 0.87 | 1.09 | 0.636 |  |
|  | Type II respiratory failure | 0.24 | 0.19 | 1.27 | 0.85 | 1.89 | 0.243 |  |
|  | LRTI | 0.08 | 0.12 | 1.08 | 0.85 | 1.38 | 0.509 |  |
|  | SCS use | -0.03 | 0.10 | 0.97 | 0.77 | 1.22 | 0.771 |  |
|  | Antibiotic treatment | -0.11 | 0.21 | 0.90 | 0.56 | 1.44 | 0.645 |  |

**Notes:**

^*^ Binary categorical variable: four or more comorbidities except for bronchiectasis (hereinafter the same).

Model 1 adjusted for confounders including gender, age, BMI, disease duration, GOLD stage, other comorbidities, smoking status, respiratory failure, AHPY, existence of LRTI; Model 2 additionally adjusted for systemic corticosteroids use and antibiotic treatment based on Model 1.

**Abbreviations:** BMI, Body mass index; SCS, Systemic corticosteroids; GOLD, Global Initiative for Chronic Obstructive Lung Disease; AHPY, AECOPD-induced hospitalizations in the past year; LRTI, Lower respiratory tract infection; OR, Odds ratio; SD, Standard deviation; CI, Confidence interval.

**Table S5 Multivariable regression analysis for the impact of bronchiectasis on secondary outcomes in AECOPD patients**

| **Outcomes** | **B** | **SD** | **OR** | **95% CI** | | ***P*-value** | **Hosmer-Lemeshow *P*-value** |
| --- | --- | --- | --- | --- | --- | --- | --- |
|  |  |  |  | **Lower limit** | **Upper limit** |  |  |
| Readmission within 30 days ^*^ | 0.00 | 0.14 | 1.00 | 0.76 | 1.33 | 0.976 | 0.186 |
| NIV ^*^ | 0.19 | 0.09 | 1.21 | 1.02 | 1.43 | 0.032 | 0.223 |
| IV ^*^ | -0.14 | 0.19 | 0.87 | 0.60 | 1.26 | 0.464 | 0.430 |
| ICU admission ^*^ | -0.37 | 0.34 | 0.69 | 0.36 | 1.34 | 0.273 | 0.389 |
| Antibiotic treatment ^‡^ | 0.81 | 0.17 | 2.25 | 1.61 | 3.15 | <0.001 | 0.653 |
| BSAT ^‡^ | 0.45 | 0.10 | 1.56 | 1.29 | 1.90 | <0.001 | 0.396 |

**Notes:**

^*^ Adjusted for covariates in Model 2.

^‡^ Adjusted for covariates in Model 1.

**Abbreviations:** ICU, intensive care unit; NIV, Non-invasive mechanical ventilation; IV, Invasive mechanical ventilation; BSAT, Broader-spectrum of antibiotic treatment. OR, Odds ratio; SD, Standard deviation; CI, Confidence interval.

**Table S6 Multivariable regression analysis for the influential factors on SCS use in total AECOPD patients and AECOPD-bronchiectasis patients**

| **Covariates** | **Total AECOPD patients *** | | | | | | **AECOPD-bronchiectasis overlap patients *** | | | | | |
| --- | --- | --- | --- | --- | --- | --- | --- | --- | --- | --- | --- | --- |
|  | **B** | **SD** | **OR** | **95% CI** | | ***P*-value** | **B** | **SD** | **OR** | **95% CI** | | ***P*-value** |
|  |  |  |  | **Lower limit** | **Upper limit** |  |  |  |  | **Lower limit** | **Upper limit** |  |
| Bronchiectasis | 0.07 | 0.06 | 1.07 | 0.95 | 1.21 | 0.254 | / | / | / | / | / | / |
| Bronchiectasis severity | / | / | / | / | / | / | -0.11 | 0.02 | 0.80 | 0.68 | 0.82 | 0.038 |
| Disease duration | 0.02 | 0.00 | 1.02 | 1.01 | 1.02 | <0.001 | 0.02 | 0.01 | 1.02 | 1.01 | 1.03 | 0.002 |
| Gender (Male) | 0.10 | 0.05 | 1.11 | 1.00 | 1.22 | 0.041 | 0.09 | 0.11 | 1.09 | 0.87 | 1.38 | 0.443 |
| Age | -0.02 | 0.00 | 0.98 | 0.98 | 0.99 | <0.001 | -0.00 | 0.01 | 1.00 | 0.99 | 1.01 | 0.517 |
| BMI | 0.00 | 0.01 | 1.00 | 0.98 | 1.02 | 0.951 | 0.02 | 0.03 | 1.02 | 0.97 | 1.08 | 0.390 |
| AHPY | 0.13 | 0.02 | 1.13 | 1.09 | 1.18 | <0.001 | 0.11 | 0.05 | 1.12 | 1.01 | 1.24 | 0.040 |
| Current smoking | -0.07 | 0.08 | 0.93 | 0.79 | 1.10 | 0.383 | -0.13 | 0.21 | 0.88 | 0.58 | 1.33 | 0.539 |
| GOLD stage | 0.06 | 0.03 | 1.22 | 0.99 | 1.31 | 0.069 | 0.08 | 0.06 | 1.25 | 1.00 | 1.35 | 0.057 |
| Comorbidity | -0.08 | 0.05 | 0.92 | 0.83 | 1.02 | 0.126 | 0.00 | 0.14 | 1.00 | 0.77 | 1.30 | 0.998 |
| Type II respiratory failure | 0.74 | 0.11 | 2.09 | 1.68 | 2.60 | <0.001 | 1.00 | 0.27 | 2.72 | 1.61 | 4.59 | <0.001 |
| LRTI | 0.03 | 0.05 | 1.03 | 0.94 | 1.13 | 0.572 | 0.24 | 0.13 | 1.27 | 0.99 | 1.63 | 0.064 |
| BEC ^‡^ | -0.00 | 0.00 | 1.00 | 0.99 | 0.99 | <0.001 | -0.00 | 0.00 | 0.99 | 0.99 | 1.00 | 0.001 |

**Notes:**

***** Hosmer-Lemeshow *P*-value was 0.191 for the analysis in AECOPD patients and 0.484 in AECOPD-bronchiectasis overlap patients.

^‡^ Absolute eosinophil numbers (cells/μL) (hereinafter the same).

**Abbreviations:** BMI, Body mass index; AHPY, AECOPD-induced hospitalizations in the past year; LRTI, Lower respiratory tract infection; GOLD, Global Initiative for Chronic Obstructive Lung Disease; SCS, Systemic corticosteroids; BEC, Blood eosinophil count; OR, Odds ratio; SD, Standard deviation; CI, Confidence interval.

**Table S7 Multivariable regression analysis for the effect of covariates on mortality in AECOPD-bronchiectasis patients**

| **Model** | **Covariates** | **B** | **SD** | **OR** | **95% CI** | | ***P*-value** | **Hosmer-Lemeshow *P*-value** |
| --- | --- | --- | --- | --- | --- | --- | --- | --- |
|  |  |  |  |  | **Lower limit** | **Upper limit** |  |  |
| 1 | Use of SCS | 0.56 | 0.33 | 1.74 | 0.91 | 3.36 | 0.097 | 0.449 |
|  | Disease duration | 0.02 | 0.01 | 1.02 | 0.99 | 1.04 | 0.153 |  |
|  | Gender (Male) | 0.09 | 0.33 | 1.09 | 0.58 | 2.07 | 0.790 |  |
|  | Age | 0.07 | 0.02 | 1.07 | 1.04 | 1.11 | <0.001 |  |
|  | BMI | -0.03 | 0.08 | 0.97 | 0.83 | 1.14 | 0.733 |  |
|  | AHPY | -0.04 | 0.15 | 0.96 | 0.72 | 1.28 | 0.780 |  |
|  | Current smoking | -0.42 | 0.63 | 0.65 | 0.19 | 2.24 | 0.500 |  |
|  | GOLD stage | 0.23 | 0.19 | 1.15 | 0.97 | 1.37 | 0.089 |  |
|  | Comorbidity | -0.45 | 0.38 | 0.64 | 0.30 | 1.34 | 0.243 |  |
|  | Type II respiratory failure | 1.43 | 0.69 | 4.17 | 1.09 | 15.98 | 0.042 |  |
|  | BEC | 0.00 | 0.00 | 1.00 | 1.00 | 1.00 | 0.934 |  |
|  | LRTI | 0.68 | 0.43 | 1.98 | 0.86 | 4.55 | 0.115 |  |
| 2 | SCS total dosage | 0.00 | 0.00 | 1.00 | 1.00 | 1.00 | 0.115 | 0.437 |
|  | Disease duration | 0.01 | 0.02 | 1.01 | 0.98 | 1.04 | 0.421 |  |
|  | Gender (Male) | 0.04 | 0.39 | 1.04 | 0.49 | 2.24 | 0.920 |  |
|  | Age | 0.06 | 0.02 | 1.07 | 1.03 | 1.11 | 0.002 |  |
|  | BMI | -0.09 | 0.11 | 0.92 | 0.7 | 1.14 | 0.437 |  |
|  | AHPY | -0.27 | 0.20 | 0.76 | 0.52 | 1.14 | 0.180 |  |
|  | Current smoking | 0.26 | 0.66 | 1.29 | 0.36 | 4.70 | 0.701 |  |
|  | Comorbidity | -0.16 | 0.44 | 0.85 | 0.36 | 2.02 | 0.713 |  |
|  | GOLD stage | 0.23 | 0.21 | 1.19 | 0.98 | 1.29 | 0.125 |  |
|  | Type II respiratory failure | 1.07 | 0.43 | 2.93 | 1.27 | 6.73 | 0.011 |  |
|  | BEC | -0.00 | 0.00 | 1.00 | 1.00 | 1.00 | 0.775 |  |
|  | LRTI | 0.70 | 0.56 | 2.01 | 0.68 | 5.98 | 0.209 |  |

**Notes:**

**Abbreviations:** BMI, Body mass index; AHPY, AECOPD-induced hospitalizations in the past year; LRTI, Lower respiratory tract infection; GOLD, Global Initiative for Chronic Obstructive Lung Disease; SCS, Systemic corticosteroids; BEC, Blood eosinophil count; OR = Odds ratio; SD, Standard deviation; CI, Confidence interval.

**Table S8 Multivariable regression analysis for the effect of SCS use and dosage on secondary outcomes in AECOPD-bronchiectasis patients ^*^**

| **Outcomes** | **SCS use** | | | | | | | **SCS dosage** | | | | | | |
| --- | --- | --- | --- | --- | --- | --- | --- | --- | --- | --- | --- | --- | --- | --- |
|  | **B** | **SD** | **OR** | **95% CI** | | ***P*-value** | **Hosmer-**  **Lemeshow *P*-value** | **B** | **SD** | **OR** | **95% CI** | | ***P*-value** | **Hosmer-**  **Lemeshow *P*-value** |
|  |  |  |  | **Lower limit** | **Upper limit** |  |  |  |  |  | **Lower limit** | **Upper limit** |  |  |
| Readmission within 30 days | 0.55 | 0.27 | 1.73 | 1.01 | 2.97 | 0.045 | 0.242 | 0.00 | 0.00 | 1.00 | 1.00 | 1.00 | 0.153 | 0.226 |
| NIV | 0.98 | 0.17 | 2.65 | 1.92 | 3.66 | <0.001 | 0.307 | 0.00 | 0.00 | 1.00 | 1.00 | 1.00 | <0.001 | 0.342 |
| IV | 0.20 | 0.36 | 1.23 | 0.61 | 2.48 | 0.569 | 0.304 | 0.00 | 0.00 | 1.00 | 1.00 | 1.00 | 0.066 | 0.503 |
| ICU admission | 0.52 | 0.71 | 1.69 | 0.42 | 6.73 | 0.459 | 0.949 | 0.00 | 0.00 | 1.00 | 1.00 | 1.00 | <0.001 | 0.966 |
| Antibiotic treatment | 2.72 | 0.61 | 15.10 | 4.55 | 50.17 | <0.001 | 0.541 | 0.02 | 0.01 | 1.02 | 1.00 | 1.04 | 0.063 | 0.878 |
| BSAT | 0.65 | 0.18 | 1.92 | 1.35 | 2.75 | <0.001 | 0.712 | 0.00 | 0.00 | 1.00 | 1.00 | 1.00 | <0.001 | 0.412 |
| LOS ^‡^ | 0.03 | 0.01 | **-** | **-** | **-** | 0.001 | **-** | 0.00 | 0.00 | **-** | **-** | **-** | <0.001 | **-** |

**Notes:**

^*^ Adjusted for covariates in Model 1.

^‡^ Dependent variable was Log_LOS. Collinear analysis: Tolerance 0.963, VIF 1.039 (SCS use); Tolerance 0.986, 1.014 (SCS dosage). The linear regression coefficient (B) is used instead of OR.

**Abbreviations:** BMI, Body mass index; AHPY, AECOPD-induced hospitalizations in the past year; LRTI, Lower respiratory tract infection; SCS, Systemic corticosteroids; OR, Odds ratio; SD, Standard deviation; NIV, Non-invasive mechanical ventilation; IV, Invasive mechanical ventilation; BSAT, Broader-spectrum of antibiotic treatment; CI, Confidence interval.

**Table S9 Characteristics of SCS-treated AECOPD-bronchiectasis patients stratified by BEC (< 300 vs. ≥ 300 cells/μL) (bafore PSM)**

| **Characteristics** | **BEC level at admission** | | ***P*-value** |
| --- | --- | --- | --- |
|  | **BEC < 300 cells/μL (n=745)** | **BEC ≥ 300 cells/μL (n=39)** |  |
| Age, year, mean (SD) | 71.55±9.94 | 70.13±8.52 | 0.468 |
| Male, n (%) | 495 (66.44) | 27 (69.23) | 0.719 |
| BMI, mean (SD) | 20.40±1.91 | 21.05±2.80 | 0.113 |
| Current cigarette smoking, n (%) | 58 (7.79) | 3 (7.69) | 0.983 |
| Disease duration, median (IQR) | 10 (10.00, 20.00) | 10 (10.00, 20.00) | 0.054 |
| AHPY, median (IQR) | 1.00 (1.00, 2.00) | 2.00 (1.00, 3.00) | 0.202 |
| Four or more comorbidities, n (%) | 178 (23.89) | 8 (20.51) | 0.629 |
| **GOLD stage, n (%)** | | | |
| I-II stage | 362 (48.59) | 26 (66.67) | 0.028 |
| III-IV stage | 383 (51.41) | 13 (33.33) |  |
| **Bronchiectasis severity, n (%)** | | |  |
| Mild | 541 (72.62) | 24 (61.54) | 0.133 |
| Moderate | 135 (18.12) | 15 (38.46) | 0.002 |
| Severe | 69 (9.26) | 0 (0.00) | 0.049 |
| **BGA at admission** | | | |
| PO2, median (IQR) | 80.50 (70.75, 92.08) | 80.50 (66.55, 99.55) | 0.700 |
| PCO2, median (IQR) | 45.80 (45.00, 59.60) | 47.60 (45.80, 57.80) | 0.436 |
| Type I respiratory failure, n (%) | 174 (23.36) | 11 (28.21) | 0.487 |
| Type II respiratory failure, n (%) | 101 (13.56) | 5 (12.82) | 0.896 |
| LRTI, n (%) | 582 (78.12) | 24 (61.54) | 0.016 |
| Mortality, n (%) | 33 (4.43) | 0 (0.00) | 0.179 |
| Readmission within 30 days, n (%) | 49 (6.58) | 1 (2.56) | 0.317 |
| **Ventilator use, n (%)** | | | |
| NIV | 177 (23.76) | 6 (15.38) | 0.228 |
| IV | 23 (3.09) | 1 (2.56) | 0.853 |
| LOS, days, median, median (IQR) | 12.00 (11.00, 12.00) | 12.00 (12.00, 13.50) | 0.112 |
| ICU admission, n (%) | 7 (0.94) | 1 (2.56) | 0.325 |
| **Medical treatment** | | | |
| Antibiotic treatment, n (%) | 742 (99.60) | 39 (100.00) | 0.691 |
| BSAT, n (%) | 115 (15.44) | 1 (2.56) | 0.027 |
| Antifungal treatment, n (%) | 83 (11.14) | 3 (7.69) | 0.502 |
| Antifungal treatment time, days, median (IQR) | 11.50 (6.00, 14.25) | 14.00 (8.50, 14.50) | 0.984 |

**Notes:**

**Abbreviations:** SD, Standard deviation; IQR, Interquartile range; BMI, Body mass index; AHPY, AECOPD-induced hospitalizations in the past year; BGA, Blood gas analysis; LRTI, Lower respiratory tract infection; BEC, Blood eosinophil count; SCS, Systemic corticosteroids; NIV, Non-invasive mechanical ventilation; IV, Invasive mechanical ventilation; BSAT, Broader-spectrum of antibiotic treatment.

**Table S10. Characteristics of SCS-treated AECOPD-bronchiectasis patients stratified by BEC (< 100, 100 –< 300, ≥ 300 cells/μL) (bafore PSM)**

| **Characteristics** | **BEC level at admission** | | | ***P*-value** |
| --- | --- | --- | --- | --- |
|  | **BEC < 100 cells/μL (n=205)** | **100 ≤ BEC < 300 cells/μL (n=540)** | **BEC ≥ 300 cells/μL (n=39)** |  |
| Age, year, mean (SD) | 71.74±11.13 | 72.70±9.14 | 70.13±8.52 | 0.469 |
| Male, n (%) | 140 (68.29) | 355 (65.74) | 27 (69.23) | 0.754 |
| BMI, mean (SD) | 20.55±1.78 | 20.13±2.19 | 21.05±2.80 | 0.397 |
| Current cigarette smoking, n (%) | 18 (8.78) | 40 (7.41) | 3 (7.69) | 0.822 |
| Disease duration, median (IQR) | 10 (10.00, 27.00) | 10 (10.00, 20.00) | 10 (10.00, 20.00) | 0.135 |
| AHPY, median (IQR) | 1.00 (1.00, 2.00) | 1.00 (1.00, 1.00) | 2.00 (1.00, 3.00) | 0.021 |
| Four or more comorbidities, n (%) | 49 (23.90) | 129 (23.89) | 8 (20.51) | 0.890 |
| **GOLD stage, n (%)** | | | | |
| I-II stage | 96 (46.83) | 266 (49.26) | 26 (66.67) | 0.075 |
| III-IV stage | 109 (53.17) | 274 (50.74) | 13 (33.33) |  |
| **BGA at admission** | | | | |
| PO2 | 80.50 (67.55, 88.20) | 80.50 (73.03, 94.50) | 80.50 (66.55, 99.55) | 0.876 |
| PCO2 | 45.80 (44.35, 58.83) | 45.80 (44.23, 60.45) | 47.60 (45.80, 57.80) | 0.893 |
| Type I respiratory failure, n (%) | 56 (27.32) | 118 (21.85) | 11 (28.21) | 0.229 |
| Type II respiratory failure, n (%) | 27 (13.17) | 74 (13.70) | 5 (12.82) | 0.974 |
| LRTI, n (%) | 168 (81.95) | 416 (92.44) | 24 (61.54) | 0.017 |
| Mortality, n (%) | 10 (4.88) | 23 (4.26) | 0 (0.00) | 0.368 |
| Readmission within 30 days, n (%) | 14 (6.83) | 35 (6.48) | 1 (2.56) | 0.598 |
| **Bronchiectasis severity, n (%)** | | | | |
| Mild | 93 (45.37) | 448 (83.00) | 24 (61.54) | <0.001 |
| Moderate | 78 (38.05) | 58 (10.74) | 15 (38.46) | <0.001 |
| Severe | 34 (16.56) | 34 (6.30) | 0 (0.00) | <0.001 |
| **Ventilator use, n (%)** | | | | |
| NIV | 44 (21.46) | 133 (24.63) | 6 (15.38) | 0.319 |
| IV | 5 (2.44) | 18 (3.33) | 1 (2.56) | 0.805 |
| LOS, days, median, median (IQR) | 12.00 (12.00, 14.00) | 12.00 (10.00, 12.00) | 12.00 (12.00, 13.50) | 0.001 |
| ICU admission, n (%) | 3 (1.46) | 4 (0.74) | 1 (2.56) | 0.420 |
| **Medical treatment** | | | | |
| Antibiotic treatment, n (%) | 204 (99.51) | 538 (99.63) | 39 (100.00) | 0.900 |
| BSAT, n (%) | 36 (17.56) | 79 (14.63) | 1 (2.56) | 0.053 |
| Antifungal treatment, n (%) | 26 (12.68) | 57 (10.56) | 3 (7.69) | 0.566 |
| Antifungal treatment time, days, median (IQR) | 11.50 (8.25, 14.00) | 11.50 (6.00, 14.25) | 14.00 (8.50, 14.50) | 0.785 |

**Notes:**

**Abbreviations:** SD, Standard deviation; IQR, Interquartile range; BMI, Body mass index; AHPY, AECOPD-induced hospitalizations in the past year; BGA, Blood gas analysis; LRTI, Lower respiratory tract infection; BEC, Blood eosinophil count; SCS, Systemic corticosteroids; NIV, Non-invasive mechanical ventilation; IV, Invasive mechanical ventilation; BSAT, Broader-spectrum of antibiotic treatment.

**Table S11 Multivariable Cox analysis for the effect of BEC on 30-day readmission in SCS-treated AECOPD-bronchiectasis patients ^*^**

| **Covariate** | **B** | **SD** | **OR** | **95% CI** | | ***P*-value** |
| --- | --- | --- | --- | --- | --- | --- |
|  |  |  |  | **Lower limit** | **Upper limit** |  |
| BEC | -0.00 | 0.00 | 0.99 | 0.99 | 1.00 | 0.025 |

**Notes:**

^*^ Adjusted for covariates in Model 1.

**Abbreviations:** BEC, Blood eosinophil count; SCS, Systemic corticosteroids; OR, Odds ratio; SD, Standard deviation; CI, Confidence interval.

**Table S12 Linear regression analysis for the effect of covariates on LOS in SCS-treated AECOPD-bronchiectasis patients ^*^**

|  | **Unstandardized coefficient** | | **Standardized coefficient** | | **t** | ***P*-value** | **95% CI of B** | | | **Collinear statistics** | |
| --- | --- | --- | --- | --- | --- | --- | --- | --- | --- | --- | --- |
|  | **B** | **SE** | **Beta** | |  |  | **Lower limit** | | **Upper limit** | **Tolerance** | **VIF** |
| BEC ^‡^ | -0.02 | 0.01 | -0.08 | -2.41 | | 0.016 | -0.05 | -0.01 | | 0.97 | 1.033 |
| Disease duration | -0.00 | 0.00 | -0.00 | -0.05 | | 0.957 | -0.00 | 0.00 | | 0.94 | 1.059 |
| Gender (Male) | -0.01 | 0.01 | -0.03 | -0.77 | | 0.440 | -0.04 | 0.02 | | 0.98 | 1.025 |
| Age | 0.00 | 0.00 | 0.05 | 1.61 | | 0.107 | 0.00 | 0.00 | | 0.95 | 1.057 |
| BMI | 0.01 | 0.00 | 0.09 | 2.84 | | 0.005 | 0.00 | 0.01 | | 0.96 | 1.044 |
| AHPY | 0.01 | 0.01 | 0.06 | 1.93 | | 0.054 | 0.00 | 0.02 | | 0.95 | 1.058 |
| Current cigarette smokin | 0.02 | 0.02 | 0.03 | 0.84 | | 0.402 | -0.03 | 0.07 | | 0.92 | 1.084 |
| Comorbidity | -0.01 | 0.02 | -0.02 | -0.70 | | 0.486 | -0.04 | 0.02 | | 0.90 | 1.109 |
| GOLD stage | 0.10 | 0.04 | 0.10 | 3.37 | | 0.061 | 0.06 | 0.15 | | 0.91 | 1.148 |
| Type II respiratory failure | 0.00 | 0.02 | 0.00 | 0.01 | | 0.993 | -0.04 | 0.04 | | 0.94 | 1.060 |
| SCS dosage | 0.00 | 0.00 | 0.27 | 7.91 | | <0.001 | 0.00 | 0.00 | | 0.88 | 1.141 |
| LRTI | -0.04 | 0.02 | -0.09 | -2.67 | | 0.008 | -0.07 | -0.01 | | 0.93 | 1.080 |
| NIV | 0.06 | 0.02 | 0.13 | 3.87 | | <0.001 | 0.03 | 0.09 | | 0.85 | 1.176 |
| IV | -0.14 | 0.04 | -0.12 | -3.63 | | <0.001 | -0.21 | -0.06 | | 0.87 | 1.155 |
| ICU admission | 0.14 | 0.07 | 0.07 | 2.04 | | 0.042 | 0.01 | 0.27 | | 0.85 | 1.177 |
| Antibiotic treatment | -0.05 | 0.10 | -0.02 | -0.54 | | 0.590 | -0.25 | 0.14 | | 0.99 | 1.014 |
| BSAT | 0.14 | 0.02 | 0.26 | 7.94 | | <0.001 | 0.11 | 0.18 | | 0.91 | 1.095 |

**Notes:**

^*^ Dependent variable was Log_LOS.

^‡^ Independent variable was categorical variable (BEC < 300 cells/μL and BEC ≥ 300 cells/μL).

**Abbreviations:** BEC, Blood eosinophil count; SCS, Systemic corticosteroids; LOS, Length of hospital stay; ICU, intensive care unit; NIV, Non-invasive mechanical ventilation; IV, Invasive mechanical ventilation; BSAT, Broader-spectrum of antibiotic treatment; OR, Odds ratio; SE, Standard error; BMI, Body mass index; AHPY, AECOPD-induced hospitalizations in the past year; LRTI, Lower respiratory tract infection; VIF, Variance inflation factor.

**Table S13 Influential effect of BEC on other outcomes in SCS-treated AECOPD-bronchiectasis patients ^*^**

| **Outcomes** | **B** | **SD** | **OR** | **95% CI** | | ***P*-value** | **Hosmer-Lemeshow**  ***P*-value** |
| --- | --- | --- | --- | --- | --- | --- | --- |
|  |  |  |  | **Lower limit** | **Upper limit** |  |  |
| NIV | 0.00 | 0.00 | 1.00 | 1.90 | 1.00 | 0.858 | 0.128 |
| IV | 0.00 | 0.00 | 1.00 | 1.00 | 1.00 | 0.753 | 0.451 |
| ICU admission | 0.00 | 0.00 | 1.00 | 1.00 | 1.01 | 0.766 | 0.970 |
| Antibiotic treatment | 0.00 | 0.01 | 1.00 | 1.00 | 1.01 | 0.844 | 0.942 |
| BSAT | -0.00 | 0.00 | 1.00 | 1.00 | 1.00 | 0.998 | 0.607 |

**Notes:**

**^*^** Adjusted for covariates in Model 1.

**Abbreviations:** BEC, Blood eosinophil count; SCS, Systemic corticosteroids; ICU, intensive care unit; NIV, Non-invasive mechanical ventilation; IV, Invasive mechanical ventilation; BSAT, Broader-spectrum of antibiotic treatment. OR, Odds ratio; SD, Standard deviation; CI, Confidence interval.
