# Supplementary figures and images for: Association between blood eosinophil count and in-hospital mortality among systemic corticosteroids-treated patients with COPD-bronchiectasis overlap: a retrospective cohort study
Source: Front Pharmacol. 2026 Apr 29;17:1781776. doi: 10.3389/fphar.2026.1781776 (PMC13167571; doi:10.3389/fphar.2026.1781776)

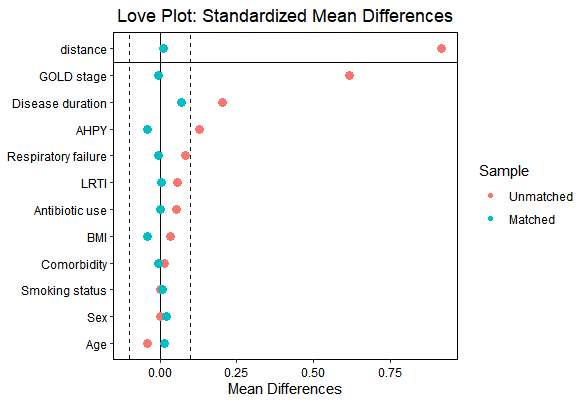

Supplement: Supplementary file 2 [file Image1.jpeg]
